# Population Perspectives on Impact of the COVID-19 Pandemic on Essential Health Services—Behavioral Insights from the Federation of Bosnia and Herzegovina

**DOI:** 10.3390/bs12120495

**Published:** 2022-12-03

**Authors:** Šeila Cilović-Lagarija, Sanjin Musa, Nino Hasanica, Goran Čerkez, Mirza Palo, Marek Majdan, Martha Scherzer, Katrine Bach Habersaat, Catherine Smallwood, Ardita Tahirukaj, Dorit Nitzan

**Affiliations:** 1 Department of Statistic, Institute for Public Health FBiH, M.Tita br.9, 71000 Sarajevo, Bosnia and Herzegovina; 2 Department of Epidemiologist, Institute for Public Health FBiH, M.Tita br.9, 71000 Sarajevo, Bosnia and Herzegovina; 3 Institute for Health and Food Safety Zenica, Institute for Public Health, Fra Ivana Franje Jukića 2, 72000 Zenica, Bosnia and Herzegovina; 4 Department for Public Health, Monitoring and Evaluation, Federal Ministry of Health, M.Tita br.9, 71000 Sarajevo, Bosnia and Herzegovina; 5 World Health Organization Country Office for Bosnia and Herzegovina, Zmaja od Bosne, 71000 Sarajevo, Bosnia and Herzegovina; 6 Department of Public Health, Faculty of Health Sciences and Social Work, Institute for Global Health and Epidemiology, Trnava University, Hornopotocna 23, 91843 Trnava, Slovakia; 7 Emergency Operations, Health Emergencies Programme, WHO Regional Office for Europe, Marmorvej 51, 2100 Copenhagen, Denmark; 8 World Health Organization Regional Office for Europe, Marmorvej 51, 2100 Copenhagen, Denmark; 9 Executive Director Division, World Health Organization Regional Office for Europe, Marmorvej 51, 2100 Copenhagen, Denmark; 10 Health Emergencies Progarmme, WHO Regional Office for Europe, Marmorvej 51, 2100 Copenhagen, Denmark

**Keywords:** COVID-19, essential health services, health systems preparedness and resilience, access to health care services

## Abstract

The aim of this study was to analyze the impact of the COVID-19 pandemic on patterns of use of essential health services (EHS), health-seeking behaviors, and population health and wellbeing in the Federation of Bosnia and Herzegovina (FBiH) from the perspective of its adult population. A population-based survey was implemented in the FBiH in December 2020 on a sample of 1068 adults. Overall, 64% of respondents received care, significantly more being women (67% vs. 61%, *p* = 0.046), those with a chronic disease (CD) (75% vs. 65%, *p* < 0.001), and of an older age (58% in 18–34 vs. 67% in older, *p* = 0.031). These groups also postponed care more often (39% in 55+ vs. 31% in 18–34 years old, *p* = 0.01; 55% with CD vs. 31% without, *p* < 0.001; and 43% in females vs. 32% males, *p* < 0.001). Main reasons for postponing care were lack of available appointments and fear of infection. The presence of a CD was the strongest predictor of need, access, and disruptions of health care. Respondents reported increased expenses for medicines (40%) and health services (30%). The findings of the survey add user insights into EHS disruptions to existing health statistics and other data and may be used to inform strategies for mitigating the impact of COVID-19 on the disruption of health care services, strengthening health system preparedness and building resilience for future emergencies.

## 1. Introduction

The COVID-19 pandemic has been associated with an immense direct impact on the health of populations globally and in the WHO European Region where by September 2022, over 250 million cases and more than 2 million deaths have been recorded [1]. Large numbers of COVID-19 patients overburdened health care systems, and the need for reorganizing health care services resulted in the disruption of many essential health services (EHSs). The second round of the national pulse survey on the continuity of EHSs during the COVID-19 pandemic, launched by WHO to better understand the extent of disruptions to EHSs globally, found that during the first quarter of 2021, 94% of the participating countries and territories reported some degree of disruptions [2].

Disruptions to EHS delivery during the COVID-19 pandemic are cause for serious concern as they are associated with excessive deaths [3,4,5,6] and have the potential for severe adverse health effects, especially among vulnerable populations [2,7]. Of all services, primary health care (PHC), rehabilitative, palliative, and long-term care were most heavily affected (40% of countries), while potentially life-saving emergency, critical, and operative care interventions were disrupted in 20% of countries [2]. Health-workforce-related reasons were the most common causes of EHS disruptions (66% of countries). Patients not presenting was reported from 57% of countries, with the most commonly reported reasons being fear of infection and mistrust (57% of countries) and financial difficulties caused by lockdowns (43% of countries) [2]. Following a period of lockdown in the Federation of Bosnia and Herzegovina (FBiH) in March 2020, most health care services were disrupted with the exception of emergency services. The combined effect of the COVID-19 pandemic and the disruptions to health care in FBiH may have led to 18% of the excessive deaths in 2020, compared to 2019 [8,9]. The extent of these disruptions and their potential impact further warrant continuous monitoring of the provision of EHSs at the country level in order to inform strategies to restore, maintain, and strengthen EHS delivery as an integral part of the response to COVID-19 and for future emergencies.

To support the countries in such an endeavor, the IMST coordination group on EHSs of the WHO regional office for Europe developed a comprehensive four-step approach to restore, maintain, and strengthen EHS provision during the COVID-19 pandemic and to improve health system preparedness for current and future emergencies. The approach consists of a rapid assessment and country situation analysis of the impact of COVID-19 on EHSs and health systems (Step 1); development of an action plan to maintain, restore, and strengthen EHS delivery during the COVID-19 pandemic (Step 2); implementation of the action plan (Step 3); and the monitoring and evaluation of the plan (Step 4).

Within the approach, a population survey on the impact of the COVID-19 pandemic on patterns of use of EHSs, including disruptions and issues of access, health-seeking behaviors, and population health and wellbeing from the community perspective was developed. Such a perspective complements the monitoring of disruptions on the level of health care providers and health facilities and brings in the perceptions from the point of view of the end user. The survey questions were integrated into an already existing survey developed by WHO regional office for Europe as a tool for countries to collect, monitor, and explore behavioral insights related to COVID-19, including demographics, protective behaviors, risk perception, wellbeing, and trust, among other variables [10].

This combined survey was conducted in the FBiH in December 2020 to comprehensively assess the behavior, access to health services, and health-seeking behavior of the adult population during the COVID-19 pandemic. The FBiH is one of the two entities which, together with the Brcko District, compose the State of Bosnia and Herzegovina (BiH). It has 2,184,680 inhabitants and consists of ten cantons with their own governments and legislatures. According to the BiH constitution law, the health care system is regulated on the entity level. The FBiH has a decentralized system which is organized on the canton level [11,12]. 

The aim of this study was to analyze the impact of the COVID-19 pandemic on patterns of use of EHSs, including disruptions and issues of access, health-seeking behaviors, and population health and wellbeing in the FBiH from the perspective of its adult population.

## 2. Methods

### 2.1. Study Design, Population, and Data Collection

In an effort to monitor the impact of the COVID-19 pandemic on access to health care and the satisfaction with care in the population of the FBiH, the Institute of Public Health of the FBiH adapted a standard COVID-19 behavioral insights questionnaire [13]. However, they also added questions from a population survey on the impact of the COVID-19 pandemic on patterns of use of EHSs, including disruptions and issues of access, health-seeking behaviors, and population health and wellbeing from the community perspective. Both standard questionnaires were provided by the WHO regional office for Europe, with the latter being part of a comprehensive four-step approach for restoring, maintaining, and strengthening EHS delivery and health system functions during COVID-19 (see Supplementary Figure S1 for more details on the four-step approach and its context). Additional questions were added from the CDC 2020 National Health Interview Survey (NHIS) Questionnaire [14]. In order to assess the patient’s satisfaction with health care, the items from the EUROPEP instrument were added (the EUROPEP tool has been previously used in the FBiH) [15,16]. The survey was translated into the Bosnian language and applied to a representative sample of 1068 respondents who were 18 years or older and residing in the FBiH in December of 2020. Data were collected by a specialized survey company, which used appropriate procedures during sampling, quota monitoring, and invitations to ensure representativeness of the population of the FBiH regarding distributions of sex, age, and geographical location. All data were collected using a system of online panels. The survey questions targeted the 9 months preceding the time of data collection, i.e., the period from March to December 2020.

### 2.2. Data Analysis

Standard data analysis for the behavioral insight surveys were conducted using the R statistical package by a team of statisticians at the University of Erfurt in Germany. In addition to descriptive statistics, linear and logistic regression analyses were conducted using backwards elimination. Confidence intervals (CI) are the 95% CI of the standardized coefficients (beta).

For the round including EHS questions, frequencies of responses were recorded and tabulated. Results are presented as frequencies of responses and as percentages overall, and are stratified by gender (male/female), residence (urban/rural), presence of chronic disease in respondent (yes/no), and age group (18–34 years, 35–54 years, and 55 years and older). For each frequency calculated, a denominator is given in the respective table—the denominators may differ based on whether the whole sample was asked to respond to the question, or whether this was only applicable for a certain subgroup (for example, the question on reasons for postponing health care was only relevant to those respondents that reported that they postponed health care and thus the denominator includes only this subset of respondents). In addition, the denominator also reflects the number of respondents in each question and group that provided a response. Satisfaction of care was analyzed by calculating overall mean scores by item and as summary, along with standard deviations—overall and stratified, as described above, with 95% confidence intervals (CI) for the whole sample and stratified, as above.

In addition to these analyses, associations between demographic and socioeconomic and health characteristics of respondents (e.g., age, gender, residence, income level, and presence of a chronic disease) and four endpoints characterizing need, barriers to access, and disruptions of health care (e.g., needed and received health care, postponement of needed health care, and increased expenses for essential medicines and for health care) were analyzed by calculating crude and adjusted odds ratios with 95% CI using logistic regression.

All analyses were performed using the R statistical language [17]. Chi-squared tests were used to test for differences in frequencies of categorical variables. Where the expected counts were below 10, Fisher’s exact test was used. To test for differences between continuous variables, the *t*-test was used. *p*-values were calculated and considered statistically significant if they were below 0.05.

### 2.3. Ethical Considerations

As an observational study with voluntary participation of the general population, the expected risk for participants was considered low. National ethical approval, as well as approval from the WHO Ethical Review Committee, were obtained before the start of the data collection. Before completing the online questionnaire, participants were duly informed and asked for their consent. Participation was voluntary, they could withdraw from participation at any time, and non-participation would not have any negative effects. Throughout the survey, the ICC/ESOMAR international code on market and social research [18] was observed and adhered to.

## 3. Results

### 3.1. Demographic Characteristics

In total, 1068 interviews were included in the analysis. The survey sample included slightly more females than males (52% vs. 48%). Residence in rural areas was reported by 37% of respondents, while 63% declared residence in urban areas. The highest percentage of respondents was in the age range between 35 and 54 years old (44%), followed by respondents under 35 years (31%) and over 54 years (25%).

#### Access to Care

**Figure 1:**
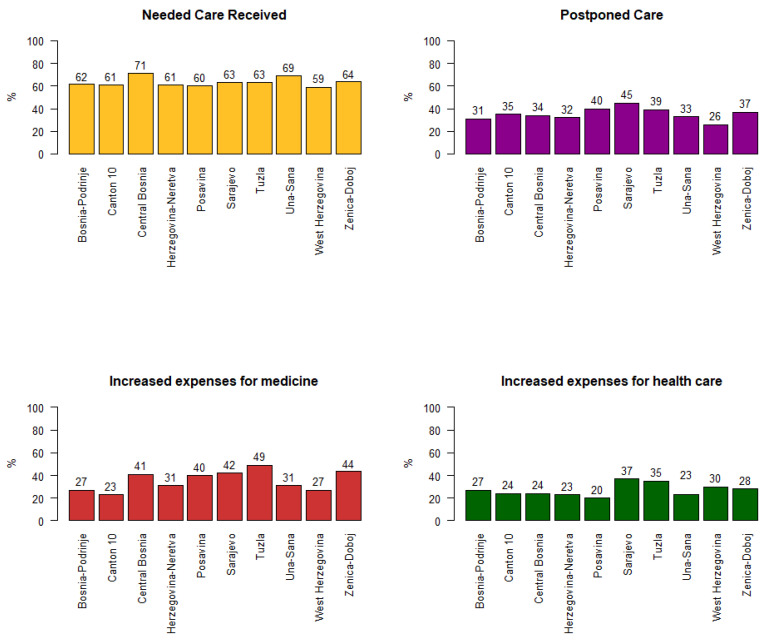
Some aspects of self-perceived access to care and postponements of care in the adult population of Bosnia and Herzegovina during the COVID-19 pandemic by region.

Overall, 64% reported that they had received the needed health care during the pandemic. This was statistically significantly higher among females (67% vs. 61% in males, *p* = 0.046, Table 1) and respondents with chronic diseases (75% vs. 61% in those without a chronic disease, *p* < 0.001). When compared by age, the youngest respondents received the needed health care significantly less frequently (58%) than respondents in the older age groups (67%, *p* = 0.031) (Table 4). In the region of West Herzegovina, less than 60% of the respondents and household members received the needed health care. The best situation was in Central Bosnia, where more than 70% of the respondents received the needed care (Figure 1).

Among the most frequent reasons for not receiving the needed care, the following were identified: inability to book an appointment with a family doctor, specialist, or hospital (54%) and postponements of visits (52%). Almost one-fifth (18%) of those who did not receive the needed care stated that the needed medications were unavailable. 

### 3.2. Out-of-Pocket Expenses for Health Care

Almost one-third of the survey participants (30%) reported higher out-of-pocket expenses for health services during the pandemic. An increase in such expenses compared to pre-COVID-19 times was observed more often by respondents from urban areas (33%), compared to those from rural areas (26%, *p* = 0.04, Table 2), those with chronic conditions (39%) compared to those without a chronic condition, *p* < 0.001, Table 3), and in older age groups (32% and 34%, *p* = 0.046, Table 4).

A similar pattern was identified for out-of-pocket expenses for medicines. Respondents with chronic diseases paid significantly more for medications during the pandemic in 53% of the cases in comparison to 35% of the cases without a chronic disease (*p* < 0.001, Table 2). Older people reported paying more for medications during the pandemic (reported by 43% in 35–54 years old and by 46% 55 years old or older), compared to those in the age group between 18 and 34 years (reported by 33%, *p* = 0.002, Table 4).

Important differences were found when comparing regions (Figure 1). An increase in out-of-pocket expenses for medicines was reported by 40% of the respondents from Posavina, 41% from Central Bosnia, 42% from Sarajevo, 44% from Zenica-Doboj, and 49% in Tuzla. In Canton 10 (23%), Bosnia-Podrinje (27%), and West Herzegovina (27%), higher expenses for medicines were declared by less than 30% of the respondents. Differences between the regions were not so apparent in the case of out-of-pocket expenses for health services.

### 3.3. Postponements of Health Care

Overall, medical care was postponed by 38% of respondents for themselves and 40% for their household members. The percentage of postponements was significantly higher among older age groups (39% and 43% vs. 31%, *p* = 0.01,Table 4), people with chronic diseases (55% vs. 31%, *p* < 0.001, Table 3), and females (43% vs. 32% in males, *p* < 0.001, Table 1). The declared percentage of postponements in health care varied by region from 26% in West Herzegovina to 45% in Sarajevo (Figure 1). The reasons most frequently given for the postponements were: the condition was not serious enough (82%), it was not possible to book an appointment or there was a long waiting time (68%), and fear of getting infected with SARS-CoV-2 (68%). The reasons were similar for household members. Dental care (38%), vision care (26%), and regular check-ups were postponed most frequently, followed by regular check-ups for hypertension (20%), cancer (17%), and diabetes (17%). Between 31% and 46% of those whose regular check-ups had been postponed were informed when the service would be available again.

### 3.4. Mental Health

Over 15% of respondents reported taking prescription medication to help with emotions, concentration, behaviour, or mental health. The percentage was statistically significantly higher among those with chronic diseases (23% vs. 12%, *p* < 0.001, Table 3) and females (17% vs. 13% in males, *p* = 0.046, Table 1). Having received counselling or therapy from a mental health professional during the pandemic was significantly lower, reported by 6% of respondents; 4% were receiving counselling or therapy at the time when the questionnaire was administered. 

### 3.5. Satisfaction with Health Care

Using a Likert scale from 1 to 5, the satisfaction with health care was assessed in 23 domains, with mean scores in each of them ranging from 2.9 for satisfaction related to disease prevention to 3.6 relating to satisfaction with emergency care and to the possibility to make appointments. The overall mean score for all 23 domains was 3.2 (95% CI: 3.2–3.3), or about 64% of the maximum possible five points. Supplemental Table S1 presents the breakdown of the scores for each of the 23 items by gender. No significant differences were found between the summary total score of satisfaction by any of the stratifications applied (e.g., gender, residence, age-group, or presence/absence of chronic disease). (See Table 5 for details).

### 3.6. Predictors of Need and Access to Health Care

Table 6 shows the results of univariate analyses of association between demographic, behavioral, socioeconomic, and health characteristics of respondents and four endpoints characterizing need for, barriers of access to, and disruptions of health care (crude odds ratios (OR) are presented). The findings suggest that the presence of a chronic disease, female gender, increasing age, higher health literacy, and higher information use, as well as trust in health professionals and institutions, were significantly associated with the reported need for health care.

The presence of chronic disease, reduced income (compared to pre-COVID-19 times), female gender, increasing age, higher information use, as well as trust in health professionals were associated with higher rates of postponement of needed health care; higher health literacy was associated with lower rates of postponements of care.

The presence of a chronic disease, worsened income, and increasing age were associated with increased expenses for medicines compared to the period before the pandemic, while respondents with increasing health literacy and trust in health institutions were less likely to report such increases in expenses. Increased expenses for health care were associated with the presence of a chronic disease, worsened income, urban residence, and increasing age; on the other hand, higher health literacy and trust in health professionals and institutions were associated with lower rates of reported increased expenses for health care during the pandemic.

**Figure 2:**
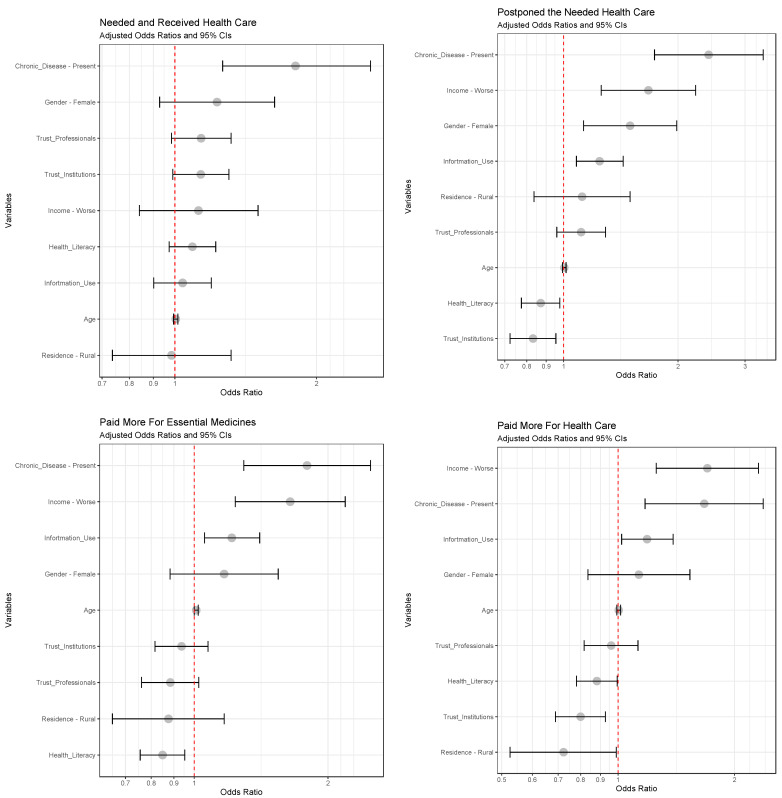
Associations between selected endpoints of barriers of access to care, postponements of care, and increased expenses for medicines and health care with relevant predictors; logistic regression adjusted odds ratios with 95% confidence intervals.

To adjust for the effects of other predictors, multivariate models were constructed. The resulting adjusted ORs with 95% CIs are shown for each model in Figure 2. These results suggest that the presence of a chronic disease independently predicts all four analyzed endpoints. Furthermore, worsened income predicted reported increased expenses for medicines and care and higher rates of care postponements. Higher information use independently predicted postponements of care and higher expenses for both medicines and care. Higher health literacy was associated with reported lower expenses for care and medicines and lower rates of care postponements. Furthermore, the association of female gender with postponements of care, and the association of trust in institutions and rural residence with increased expenses for care, also remained significant.

## 4. Discussion

### 4.1. Main Findings

A cross-sectional, population-based survey aiming to analyze the impact of the COVID-19 pandemic on patterns of use of EHSs, including disruptions and issues of access, health-seeking behaviors, and population health and wellbeing in the FBiH from the perspective of its adult population was conducted. The results showed that, overall, 64% of the respondents needed and received health care between March and December 2020. Care was needed significantly more among women (67% compared to 61% in men), in those with a chronic disease (75% vs. 65% without), and in older age groups (58% in 18–34 years old vs. 67% on older). Similarly, these groups have had their medical care postponed significantly more often, which suggests that they are most vulnerable to EHS disruptions. In the adjusted analyses, the presence of a chronic disease has been identified as the strongest independent predictor of need, access, and disruptions of health care in all models. In addition, reported worsened personal income during the pandemic (compared to the pre-pandemic period) was associated with reported increased expenses for medicines and health care. As the main reasons for postponement, no available appointment or fear of infection were reported (both by 68% of respondents). Increased expenses for medicines and health services were reported in 40%, and 30% of respondents, respectively.

### 4.2. Interpretation and Comparison with Published Literature

BiH administratively consists of and is governed as two independent political entities—the Republika Srpska (RS) and the FBiH (in which the presented survey was implemented), and one autonomous district—the Brcko District of BiH [19]. In the FBiH, each of the cantons has its own decentralized system. The reform of the health care system started in 1995 with the main aim of strengthening PHC to include family medicine. The health system in the country is complex, and this posed a challenge when organizing a response to the COVID-19 pandemic [20,21]. The universal health coverage (UHC) index, which aims to represent service coverage across population health needs and the extent to which these services could contribute to improve health, was estimated at 64.2 in 2019; while this is an increase from 54.2 in 1990, it is still below the value for most Western European countries (above 80) [22].

One of the key challenges in the provision of health care in the FBiH is the shortage of health care workers (HCWs). According to a recent analysis [11], while the number of medical doctors has been increasing in recent years, it is still below the EU average (232 vs. 353 MDs per 100,000) [23]. Similarly, the number of hospital beds available before the pandemic were considerably lower than the EU average (370 vs. 553/100,000) [12]. Such shortages may pose challenges in cases of health emergencies, and they may be a contributing factor to the findings that non-availability of appointments and long waiting times were among main reasons for postponements of care (reported by 68% of respondents). Indeed, this finding is in line with the generally high number of countries reporting HCW-related disruptions (e.g., staff shortages, infections among HCWs, etc.) as a cause of disrupted EHSs during the COVID-19 pandemic (66% of countries) [2]. In addition, HCWs are among the most vulnerable to COVID-19 [24,25] and are prone to severe burdens on mental health caused by work-related stress during the pandemic [26,27], which can further disrupt the available capacities. Therefore, addressing HCW shortages is a critical strategy in increasing preparedness and resilience for future emergencies. The WHO outlines the domains that require attention to address these shortages, including supporting and protecting HCWs at the workplace, strengthening and optimizing HCW teams, increasing capacity and strategic HCW deployment, and health system human resources strengthening through assessment and planning of HCW needs or strengthening governance and intersectoral collaboration mechanisms [28]. WHO policy considerations also provide evidence-based strategies for engaging and motivating HCWs [29]. The CDC divides strategies into contingency (e.g., adjusting staff schedules) and crisis-capacity strategies to be implemented after contingency strategies (e.g., implementing regional plans to transfer patients with COVID-19 to designated institutions) [30].

In the survey, people with chronic conditions needed care more often than those without and can thus be considered more vulnerable to any disruptions of EHS. Moreover, the presence of chronic disease reported by the respondents in the survey was found to be the single strongest factor associated with need of care, with postponement of the needed care, and with reported increased expenses for medicines and health care. This is of significant concern, as these conditions (such as ischemic heart disease, stroke, cancer, and diabetes) are the leading cause of death and disability in BiH [31]. Adding to this significance is the fact that disruptions of non-communicable disease (NCD)-related health services due to the COVID-19 pandemic were seen globally in 75% of countries. In addition, countries were shifting funds from NCD-related budgets to fund the COVID-19 response, which further disrupted these services [32]. There is strong evidence that some NCDs are risk factors for severe COVID-19 [33], making one-fifth of the global population susceptible to such events [34]. Thus, there is a two-way relationship between NCDs and COVID-19 that has been observed globally [35,36,37] and has been confirmed by the findings of this study for the FBiH. This new dimension of NCD prevalence and care should be considered in efforts to prevent and improve care for NCDs in the country. A recent report by the NCD coalition [38] provided good practices for better integrating NCD care into the COVID-19 response and beyond, for example by expanding the use of telehealth consultations, encouraging home visits by community health care workers, supporting home care, introducing multi-month prescriptions, and making medicine refills easier. Another study presented solutions to improve access to medication for people with NCDs and improve NCD care in general, including the use of remote options for prescriptions, authorizing a wider range of professionals to prescribe medication, creating electronic systems to support patients in long-term treatment, and encouraging patient empowerment and patient-centered care [39]. Furthermore, the COVID-19 pandemic has emphasized the need for a supply chain that can quickly adjust to changing needs through strategies such as regular evaluation of critical supply stock, ensuring longer-term stockpiles, improving storage facilities, and improving supplier monitoring [40]. It is important that measures and improvements implemented during the COVID-19 pandemic are maintained so that health systems are strengthened and more resilient for future emergencies.

The behavioral insights survey found reported increases in unhealthy behaviors relating to use of tobacco andalcohol, diet, and physical exercise, which are considered risk factors for NCDs. When asked about their behaviors during the last two weeks compared with pre-pandemic behaviors, 38% reported having exercised less than usual, 21% reported having eaten unhealthier food, and 6% reported having been drinking more alcohol [41]. These behaviors are generally more common among respondents who have experienced worsening financial situations. Quarantine was associated with less exercise and more unhealthy food specifically. Besides health care, it is crucial to consider the broader implications of pandemic response interventions on population health, as well as to uphold the support for healthy behaviors for children and adults and maintain population-level interventions for these risk factors.

Along with people with chronic conditions, women and the elderly were identified in this study as being among those most in need of care, and therefore most vulnerable to EHS disruptions. This is in line with findings from previous studies. Women (compared to men) were about 1.3 times, and the elderly between 65 and 75 years were 1.4 times more likely (compared to 45–54 years old) to experience EHS disruptions [42]. Women, in addition, are especially more vulnerable due to their need for specific types of health care, such as maternal or neonatal care, which were also shown to be disrupted [43]. Such disruptions can in turn increase the occurrence of related undesired health outcomes (e.g., stillbirths, maternal deaths, ruptured ectopic pregnancies, and higher maternal depression rates) [44,45], especially in countries with lower incomes [46]. The elderly, as a vulnerable group, are of great concern, as the population structure of BiH is projected to shift towards older age groups [47]. The elderly have been shown to be prone to experiencing EHS disruptions in general [46,48], and especially in hospital care [46]. Thus, both the evidence from this survey and the mounting evidence from the published literature call for attention to these two population groups and for efforts to ensure their access to EHSs during emergencies such as the COVID-19 pandemic. Specifically for increasing consumption of alcohol during the pandemic, men were found to be at higher risk.

Another significant finding is that 41% of respondents reported increased out-of-pocket payments for medicines, and 30% reported such an increase for health-service-related payments. Although a health insurance system in the FBiH is well established, it is fragmented—there are in total 13 health insurance funds, and reimbursement policies vary between them [21]. As a result, for example, inequities existed in access to essential medicines for inhabitants living in different administrative regions, even before the pandemic [49]. The problem is not isolated to the FBiH. A recent WHO report stated that out-of-pocket payments remain the dominant source of health care financing in most lower-middle-income and in about a third of upper-middle-income countries and called for reducing these payments to progress towards UHC [50]. Another study has linked COVID-19 mortality with out-of-pocket expenditure in general [51]. While based on the data obtained in this survey it is not possible to directly make such link for the FBiH, the COVID-19-related mortality in the country has been shown to be among the highest overall in the global context [47]. Therefore, efforts should be made to mitigate the problem and recognize it as one of the possible drivers of undesired health outcomes during the COVID-19 pandemic. In general, COVID-19 has placed health as a strategic sector in the economy and put additional emphasis on the ability quickly to adjust to changing needs of the population.

The survey also assessed the satisfaction with health care in 23 domains and found an average satisfaction score of 3.2 (95% CI: 3.2–3.3). While it is difficult to interpret these findings in absolute terms, a comparison with similar surveys in one of the regions of the FBiH in 2011 (yielding a mean score 3.2, range 2.6–3.8) and in 2017 (yielding a mean score of 3.5, range 3.1–3.9) [15] suggests that, due to the pandemic, the satisfaction with health care during the COVID-19 pandemic declined from 2017 levels to levels similar to those measured in 2011. An in-depth study is needed to elaborate on these differences. However, based on these comparisons, the COVID-19 pandemic caused a decline in overall patient satisfaction with care in the country.

We note that since the data collection for this study took place, the numbers of cases of COVID-19 have declined throughout Europe, including in the FBiH [1], which led to the gradual lifting of restrictions of movement and reinstalment of most services to pre-pandemic levels [51]. This, in turn, likely led to fewer disruptions of EHSs, to the mitigation of barriers of access to care and health-seeking behavior, and to the general improvement of health care service availability and use. Despite these improvements, the findings of this study present important lessons that can be used to avoid disruptions of EHSs during future emergencies, as well as to improve resilience and preparedness for such events.

### 4.3. Study Limitations

The study relies on data from a population-based online survey. The findings reflect self-perceived and self-reported characteristics, which may result in a reporting bias. Using online web panels may produce bias against people without access to an internet connection, computers, smart phones, and other digital devices, including potentially the elderly population and disadvantaged population groups. However, the panels used included people in all age groups, and a concerted effort was made to ensure inclusion of the elderly age group. We note that all efforts were made to overcome these biases by applying a valid and sophisticated sampling strategy and in all steps of the implementation of the survey and analysis of its findings. Thus, despite these limitations, the study presents very valuable findings that describe the views and perceptions of access to EHSs by the adult population of BiH during a period of the COVID-19 health emergency. The survey tool used questions from four different questionnaires, already validated. While this may be a potential limitation, it can also be considered a strength, as this setup allowed for regression analysis across the various factors and helped us to understand how other variables affect the disruptions of EHSs.

## 5. Conclusions

This study provides an important snapshot of the impact of the COVID-19 pandemic on patterns of use of the EHSs, including disruptions and issues of access, health-seeking behaviors, and population health and wellbeing in the FBiH from the perspective of its adult population. These findings provide valuable insights into the indirect and associated effects of the pandemic response, which is likely to be similar beyond the FBiH, potentially in every country where lockdowns were introduced, and the health system redirected to respond to the pandemic. This adds important user insights into EHS disruptions assessed based on health statistics or by health care providers. The findings of the survey should inform strategies for mitigating the impact of COVID-19 on the disruption of health care and broader health promotion, as well as strategies for strengthening health systems and increasing their resilience for future emergencies in the FBiH and beyond.

## Figures and Tables

**Table 1:** Self-perceived access to care, postponements of care, and mental health in the adult population of Bosnia and Herzegovina during the COVID-19 pandemic by sex.

Variable Name	Sex of Respondents (n, %)			
	Males 514 (48%)	Females 554 (52%)	Total 1068	*p*-Value
Access to health care				
Needed and received care (n, % Yes)	314/514 (61%)	372/554 (67%)	686/1068 (64%)	0.046
Way of not getting needed care (n, % Yes)				
Postponed visit to the family doctor	100/200 (50%)	97/182 (53%)	197/382 (52%)	0.588
Postponed planned surgery	32/200 (16%)	27/182 (15%)	59/382 (15%)	0.863
Not able to book an appointment at family doctor, specialist, hospital	107/200 (54%)	99/182 (54%)	206/382 (54%)	0.942
Needed medication was not available	36/200 (18%)	33/182 (18%)	69/382 (18%)	1
Other	65/200 (33%)	57/182 (31%)	122/382 (32%)	0.891
Increased out-of-pocket expenses for medicines (n, % Yes)	188/483 (39%)	225/530 (42%)	413/1013 (41%)	0.281
Increased out-of-pocket expenses for health services (n, % Yes)	137/479 (29%)	166/523 (32%)	303/1002 (30%)	0.312
Postponements of care				
Postponed medical care (n, % Yes)	165/514 (32%)	239/554 (43%)	404/1068 (38%)	<0.001
Main reason for postponement (n, % Yes) *				
Condition was not serious enough	129/165 (78%)	202/239 (85%)	331/404 (82%)	0.135
No appointment/long waiting time	122/165 (74%)	154/239 (64%)	276/404 (68%)	0.056
Facility was closed	67/165 (41%)	70/239 (29%)	137/404 (34%)	0.024
Fear of infection with SARS-CoV-2	108/165 (65%)	168/239 (70%)	276/404 (68%)	0.358
Other	42/165 (25%)	47/239 (20%)	89/404 (22%)	0.208
Mental health				
Taking prescription medication to help with emotions, concentration, behavior, or mental health (n, % Yes)	63/493 (13%)	94/539 (17%)	157/1032 (15%)	0.046
Receiving counselling or therapy from a mental health professional in past 9 months (n, % Yes)	30/498 (6%)	33/541 (6%)	63/1039 (6%)	1
Currently receiving counselling or therapy from a mental health professional (n, % Yes)	22/496 (4%)	22/544 (4%)	44/1040 (4%)	0.874

* Some denominators differ from the sample in the respective group based on whether the question was applicable to that group or based on the number of potential respondents that provided a response to the question.

**Table 2:** Self-perceived access to care, postponements of care, and mental health in the adult population of Bosnia and Herzegovina during the COVID-19 pandemic by residence of respondents *.

Variable Name	Residence of Respondents (n, %)			
	Rural 398 (37%)	Urban 670 (63%)	Total 1068	*p*-Value
Access to health care				
Needed and received care (n, % Yes)	258/398 (65%)	428/670 (64%)	686/1068 (64%)	0.806
Way of not getting needed care (n, % Yes)				
Postponed visit to the family doctor	70/140 (50%)	127/242 (52%)	197/382 (52%)	0.718
Postponed planned surgery	16/140 (11%)	43/242 (18%)	59/382 (15%)	0.132
Not able to book an appointment at family doctor, specialist, hospital	76/140 (54%)	130/242 (54%)	206/382 (54%)	1
Needed medication was not available	18/140 (13%)	51/242 (21%)	69/382 (18%)	0.061
Other	47/140 (34%)	75/242 (31%)	122/382 (32%)	0.684
Increased out-of-pocket expenses for medicines (n, % Yes)	149/379 (39%)	264/634 (42%)	413/1013 (41%)	0.507
Increased out-of-pocket expenses for health services (n, % Yes)	99/377 (26%)	204/625 (33%)	303/1002 (30%)	0.040
Postponements of care				
Postponed medical care (n, % Yes)	156/398 (39%)	248/670 (37%)	404/1068 (38%)	0.519
Main reason for postponement (n, % Yes)				
Condition was not serious enough	125/156 (80%)	206/248 (83%)	331/404 (82%)	0.539
No appointment/long waiting time	104/156 (67%)	172/248 (69%)	276/404 (68%)	0.649
Facility was closed	57/156 (37%)	80/248 (32%)	137/404 (34%)	0.437
Fear of infection with SARS-CoV-2	104/156 (67%)	172/248 (69%)	276/404 (68%)	0.649
Other	31/156 (20%)	58/248 (23%)	89/404 (22%)	0.480
Mental health				
Taking prescription medication to help with emotions, concentration, behavior, or mental health (n, % Yes)	48/382 (13%)	109/650 (17%)	157/1032 (15%)	0.084
Receiving counselling or therapy from a mental health professional in past 9 months (n, % Yes)	22/390 (6%)	41/649 (6%)	63/1039 (6%)	0.758
Currently receiving counselling or therapy from a mental health professional (n, % Yes)	18/389 (5%)	26/651 (4%)	44/1040 (4%)	0.740

* Some denominators differ from the sample in the respective group based on whether the question was applicable to that group or based on the number of potential respondents that provided a response to the question.

**Table 3:** Self-perceived access to care, postponements of care, and mental health in the adult population of Bosnia and Herzegovina during the COVID-19 pandemic by presence of chronic diseases *.

Variable Name	Chronic Disease Present (n, %)			
	No 731 (76%)	Yes 237 (24%)	Total 968	*p*-Value
Access to health care				
Needed and received care (n, % Yes)	447/731 (61%)	178/237 (75%)	625/968 (65%)	<0.001
Way of not getting needed care (n, % Yes)				
Postponed visit to the family doctor	138/284 (49%)	37/59 (63%)	175/343 (51%)	0.067
Postponed planned surgery	41/284 (14%)	10/59 (17%)	51/343 (15%)	0.770
Not able to book an appointment at family doctor, specialist, hospital	141/284 (50%)	44/59 (75%)	185/343 (54%)	<0.001
Needed medication was not available	47/284 (17%)	12/59 (20%)	59/343 (17%)	0.608
Other	89/284 (31%)	19/59 (32%)	108/343 (31%)	1
Increased out-of-pocket expenses for medicines (n, % Yes)	245/691 (35%)	124/233 (53%)	369/924 (40%)	<0.001
Increased out-of-pocket expenses for health services (n, % Yes)	184/690 (27%)	85/220 (39%)	269/910 (30%)	<0.001
Postponements of care				
Postponed medical care (n, % Yes)	229/731 (31%)	130/237 (55%)	359/968 (37%)	<0.001
Main reason for postponement (n, % Yes)				
Condition was not serious enough	199/229 (87%)	96/130 (74%)	295/359 (82%)	0.003
No appointment/long waiting time	152/229 (66%)	92/130 (71%)	244/359 (68%)	0.459
Facility was closed	74/229 (32%)	49/130 (38%)	123/359 (34%)	0.360
Fear of infection with SARS-CoV-2	155/229 (68%)	87/130 (67%)	242/359 (67%)	0.975
Other	47/229 (21%)	30/130 (23%)	77/359 (21%)	0.665
Mental health				
Taking prescription medication to help with emotions, concentration, behavior, or mental health (n, % Yes)	87/713 (12%)	53/229 (23%)	140/942 (15%)	<0.001
Receiving counselling or therapy from a mental health professional in past 9 months (n, % Yes)	35/711 (5%)	20/235 (9%)	55/946 (6%)	0.061
Currently receiving counselling or therapy from a mental health professional (n, % Yes)	27/710 (4%)	13/235 (6%)	40/945 (4%)	0.34

* Some denominators differ from the sample in the respective group based on whether the question was applicable to that group or based on the number of potential respondents that provided a response to the question.

**Table 4:** Self-perceived access to care, postponements of care, and mental health in the adult population of Bosnia and Herzegovina during the COVID-19 pandemic by age groups *.

Variable Name	Age Group of Respondents (n, %)				
	18–34 Years 327 (31%)	35–54 Years 473 (44%)	55+ Years 268 (25%)	Total 1068	*p*-Value
Access to health care					
Needed and received care (n, % Yes)	191/327 (58%)	316/473 (67%)	179/268 (67%)	686/1068 (64%)	0.031
Way of not getting needed care (n, % Yes)					
Postponed visit to the family doctor	64/136 (47%)	90/157 (57%)	43/89 (48%)	197/382 (52%)	0.168
Postponed planned surgery	21/136 (15%)	25/157 (16%)	13/89 (15%)	59/382 (15%)	0.963
Not able to book an appointment at family doctor, specialist, hospital	61/136 (45%)	91/157 (58%)	54/89 (61%)	206/382 (54%)	0.028
Needed medication was not available	26/136 (19%)	21/157 (13%)	22/89 (25%)	69/382 (18%)	0.078
Other	32/136 (24%)	52/157 (33%)	38/89 (43%)	122/382 (32%)	0.010
Increased out-of-pocket expenses for medicines (n, % Yes)	99/303 (33%)	196/453 (43%)	118/257 (46%)	413/1013 (41%)	0.002
Increased out-of-pocket expenses for health services (n, % Yes)	76/305 (25%)	141/442 (32%)	86/255 (34%)	303/1002 (30%)	0.046
Postponements of care					
Postponed medical care (n, % Yes)	103/327 (31%)	185/473 (39%)	116/268 (43%)	404/1068 (38%)	0.010
Main reason for postponement (n, % Yes)					
Condition was not serious enough	88/103 (85%)	154/185 (83%)	89/116 (77%)	331/404 (82%)	0.202
No appointment/long waiting time	71/103 (69%)	122/185 (66%)	83/116 (72%)	276/404 (68%)	0.589
Facility was closed	42/103 (41%)	61/185 (33%)	34/116 (29%)	137/404 (34%)	0.189
Fear of infection with SARS-CoV-2	72/103 (70%)	126/185 (68%)	78/116 (67%)	276/404 (68%)	0.912
Other	22/103 (21%)	35/185 (19%)	32/116 (28%)	89/404 (22%)	0.207
Mental health					
Taking prescription medication to help with emotions, concentration, behavior, or mental health (n, % Yes)	37/314 (12%)	74/459 (16%)	46/259 (18%)	157/1032 (15%)	0.108
Receiving counselling or therapy from a mental health professional in past 9 months (n, % Yes)	20/312 (6%)	32/466 (7%)	11/261 (4%)	63/1039 (6%)	0.340
Currently receiving counselling or therapy from a mental health professional (n, % Yes)	14/307 (5%)	20/469 (4%)	10/264 (4%)	44/1040 (4%)	0.900

* Some denominators differ from the sample in the respective group based on whether the question was applicable to that group or based on the number of potential respondents that provided a response to the question.

**Table 5:** Satisfaction with health care in the adult population of Bosnia and Herzegovina during the COVID-19 pandemic by selected aspects.

Score	Gender		Residence		Age Group			Chronic Disease		Total
	Males	Females	Urban	Rural	18–34	35–54	55+	YES	NO	
Mean Score (95% CI)	3.2 (3.1–3.3)	3.2 (3.1–3.4)	3.2 (3.1–3.4)	3.2 (3.1–3.3)	3.2 (3.1–3.4)	3.2 (3.1–3.3)	3.2 (3.1–3.4)	3.3 (3.1–3.4)	3.3 (3.1–3.4)	3.2 (3.2–3.3)
*p*-value	0.745		0.810		0.947			0.936		

**Table 6:** Associations between selected endpoints of barriers of access to care, postponements of care, and increased expenses for medicines and health care with relevant predictors; crude odds ratios with 95% confidence intervals.

Predictor	Needed and Received Health Care		Postponed the Needed Health Care		Increased Expenses for Essential Medicines		Increased Expenses for Health Care	
	OR (95% CI)	*p*-value	OR (95% CI)	*p*-Value	OR (95% CI)	*p*-value	OR (95% CI)	*p*-Value
Age	1.01 (1.002–1.02)	0.016	1.01 (1.002–1.02)	<0.01	1.01 (1.01–1.02)	<0.01	1.01 (1.004–1.02)	0.04
Sex								
Female (vs. Male)	1.3 (1.03–1.7)	0.039	1.6 (1.2–2.1)	<0.001	1.2 (0.9–1.5)	0.254	1.2 (0.9–1.5)	0.28
Education								
Primary/HS (vs. College)	1.08 (0.81–1.44)	0.569	0.85 (0.64–1.13)	0.282	0.83 (0.62–1.1)	0.205	0.51 (0.38–0.69)	<0.001
Residence								
Rural (vs. Urban)	1.04 (0.8–1.35)	0.756	1.1 (0.84–1.4)	0.477	0.91 (0.7–1.2)	0.466	0.73 (0.55–0.98)	0.035
Income								
Worse (vs. Same/improved)	1.09 (0.78–1.31)	0.946	1.8 (1.4–2.3)	<0.001	1.9 (1.5–2.5)	<0.001	1.8 (1.4–2.4)	<0.001
Chronic disease								
Present (vs. No)	1.91 (1.38–2.66)	<0.001	2.7 (2–3.6)	<0.001	2.1 (1.5–2.8)	<0.001	1.7 (1.3–2.4)	<0.001
Health Literacy								
Point increase (7-point scale)	1.2 (1.1–1.4)	<0.001	0.9 (0.82–0.98)	0.025	0.85 (0.77–0.93)	<0.01	0.82 (0.74–0.9)	<0.001
Information Use								
Point increase (7-point scale)	1.2 (1.1–1.4)	<0.001	1.1 (1.04–1.2)	<0.01	1.03 (0.96–1.1)	0.393	0.97 (0.89–1.06)	0.484
Trust in Professionals								
Point increase (7-point scale)	1.3 (1.2–1.4)	<0.001	1.08 (1.01–1.2)	0.032	0.95 (0.88–1.03)	0.251	0.92 (0.84–0.99)	0.038
Trust in Institutions								
Point increase (8-point scale)	1.3 (1.2–1.4)	<0.001	0.95 (0.88–1.04)	0.302	0.92 (0.85–0.99)	0.047	0.82 (0.75–0.9)	<0.001

OR = odds ratio; CI = confidence interval; HS = high school.

## Data Availability

Data are available from the lead author upon reasonable request.

## Supplementary Materials

The following supporting information can be downloaded at: https://www.mdpi.com/article/10.3390/bs12120495/s1, Supplemental Table S1: Satisfaction with health care in the adult population of Bosnia and Herzegovina during the COVID-19 pandemic by sex, Supplementary Figure S1: Outline of the sequence of steps and context of the Four step approach to maintain, restore and strengthen the provision of EHS during the COVID-19 and to increase the preparedness and resilience of health systems for future emergencies.

## Open Access Statement

In accordance with WHO’s open-access publication policy for all work funded by WHO or authored/co-authored by WHO staff members, the WHO retains the copyright of this publication through a Creative Commons Attribution IGO license (http://creativecommons.org/licenses/by/3.0/igo/legalcode accessed on 12 January 2021) which permits unrestricted use, distribution, and reproduction in any medium provided the original work is properly cited.
