# Supplementary material for: Population Perspectives on Impact of the COVID-19 Pandemic on Essential Health Services—Behavioral Insights from the Federation of Bosnia and Herzegovina
Source: Behav Sci (Basel). 2022 Dec 3;12(12):495. doi: 10.3390/bs12120495 (PMC9774738; doi:10.3390/bs12120495)

behavioral sciences

MDPI

Supplemental Table S1: Satisfaction with health care in the adult population of Bosnia and Herzegovina during the COVID-19 pandemic by sex.

Question	SEX OF RESPONDENTS (mean ± SD)			
	males	females	total	p-value
Do you think that the family doctor spends sufficient time with you?	2.9 ± 1.4 (n = 468)	3.1 ± 1.5 (n = 497)	3.0 ± 1.4 (n = 965)	0.131
Does the doctor show any interest in your problem?	3.2 ± 1.4 (n = 456)	3.3 ± 1.4 (n = 495)	3.2 ± 1.4 (n = 951)	0.355
Do you feel better when you tell the doctor about your problem?	3.4 ± 1.4 (n = 465)	3.6 ± 1.3 (n = 496)	3.5 ± 1.4 (n = 961)	0.017
Does the doctor involve you in making a decision about your treatment?	3.4 ± 1.3 (n = 462)	3.4 ± 1.3 (n = 497)	3.4 ± 1.3 (n = 959)	0.910
Does the doctor listen to you carefully while you are presenting your problems?	3.5 ± 1.4 (n = 468)	3.5 ± 1.4 (n = 508)	3.5 ± 1.4 (n = 976)	0.917
Does the doctor provide you with all information about your diseases?	3.4 ± 1.3 (n = 467)	3.4 ± 1.4 (n = 505)	3.4 ± 1.3 (n = 972)	0.888
Does the doctor try hard to relieve your symptoms as soon as possible?	3.5 ± 1.3 (n = 467)	3.5 ± 1.3 (n = 502)	3.5 ± 1.3 (n = 969)	0.569
Does the doctor help you feel better and return to your everyday work?	3.4 ± 1.3 (n = 464)	3.5 ± 1.3 (n = 497)	3.5 ± 1.3 (n = 961)	0.788
Does the doctor perform a physical examination?	3.6 ± 1.3 (n = 456)	3.5 ± 1.4 (n = 489)	3.5 ± 1.4 (n = 945)	0.130
Does the doctor perform a detailed physical examination?	3.3 ± 1.4 (n = 459)	3.3 ± 1.4 (n = 502)	3.3 ± 1.4 (n = 961)	0.434
Does the doctor work on the prevention of various diseases?	3.0 ± 1.4 (n = 453)	2.9 ± 1.4 (n = 485)	2.9 ± 1.4 (n = 938)	0.513
Does the doctor explain why you need to undergo additional tests and analyses?	3.3 ± 1.4 (n = 453)	3.3 ± 1.4 (n = 497)	3.3 ± 1.4 (n = 950)	0.529
Does the doctor provide explanation about your symptoms and disease?	3.4 ± 1.3 (n = 454)	3.4 ± 1.4 (n = 492)	3.4 ± 1.4 (n = 946)	0.974
Does the doctor help with your emotional problems related to your health condition?	3.0 ± 1.4 (n = 446)	3.0 ± 1.4 (n = 479)	3.0 ± 1.4 (n = 925)	0.771
Does the doctor explain to you why it is important to comply with his/her instructions?	3.4 ± 1.3 (n = 459)	3.4 ± 1.4 (n = 499)	3.4 ± 1.3 (n = 958)	0.997
Does the doctor explain what he/she is doing during the examination?	3.3 ± 1.3 (n = 455)	3.2 ± 1.4 (n = 495)	3.3 ± 1.4 (n = 950)	0.380
Does the doctor explain what you can expect at a specialist examination in hospital?	3.1 ± 1.3 (n = 453)	3.0 ± 1.4 (n = 495)	3.0 ± 1.4 (n = 948)	0.430
Are you assisted by other medical staff (nurse at the clinic)?	3.5 ± 1.3 (n = 460)	3.3 ± 1.3 (n = 504)	3.4 ± 1.3 (n = 964)	0.175
Can you make an appointment with the doctor?	3.6 ± 1.3 (n = 477)	3.7 ± 1.4 (n = 510)	3.6 ± 1.3 (n = 987)	0.396
Is it easy to make a phone call to the doctor?	3.0 ± 1.5 (n = 476)	3.0 ± 1.5 (n = 517)	3.0 ± 1.5 (n = 993)	0.4
Can you seek advice from the doctor by phone?	3.0 ± 1.5 (n = 469)	3.0 ± 1.5 (n = 515)	3.0 ± 1.5 (n = 984)	0.742
Do you wait long in the waiting room?	3.3 ± 1.4 (n = 457)	3.3 ± 1.4 (n = 509)	3.3 ± 1.4 (n = 966)	0.921
Does the doctor respond fast in emergency situation?	3.5 ± 1.3 (n = 451)	3.6 ± 1.3 (n = 494)	3.6 ± 1.3 (n = 945)	0.305
TOTAL SCORE	74.6 ± 24.6	74.0 ± 24.5	74.3 ± 24.5	0.745

Supplementary Figure S1: Outline of the sequence of steps and context of the Four step approach to maintain, restore and strengthen the provision of EHS during the COVID-19 and to increase the preparedness and resilience of health systems for future emergencies.

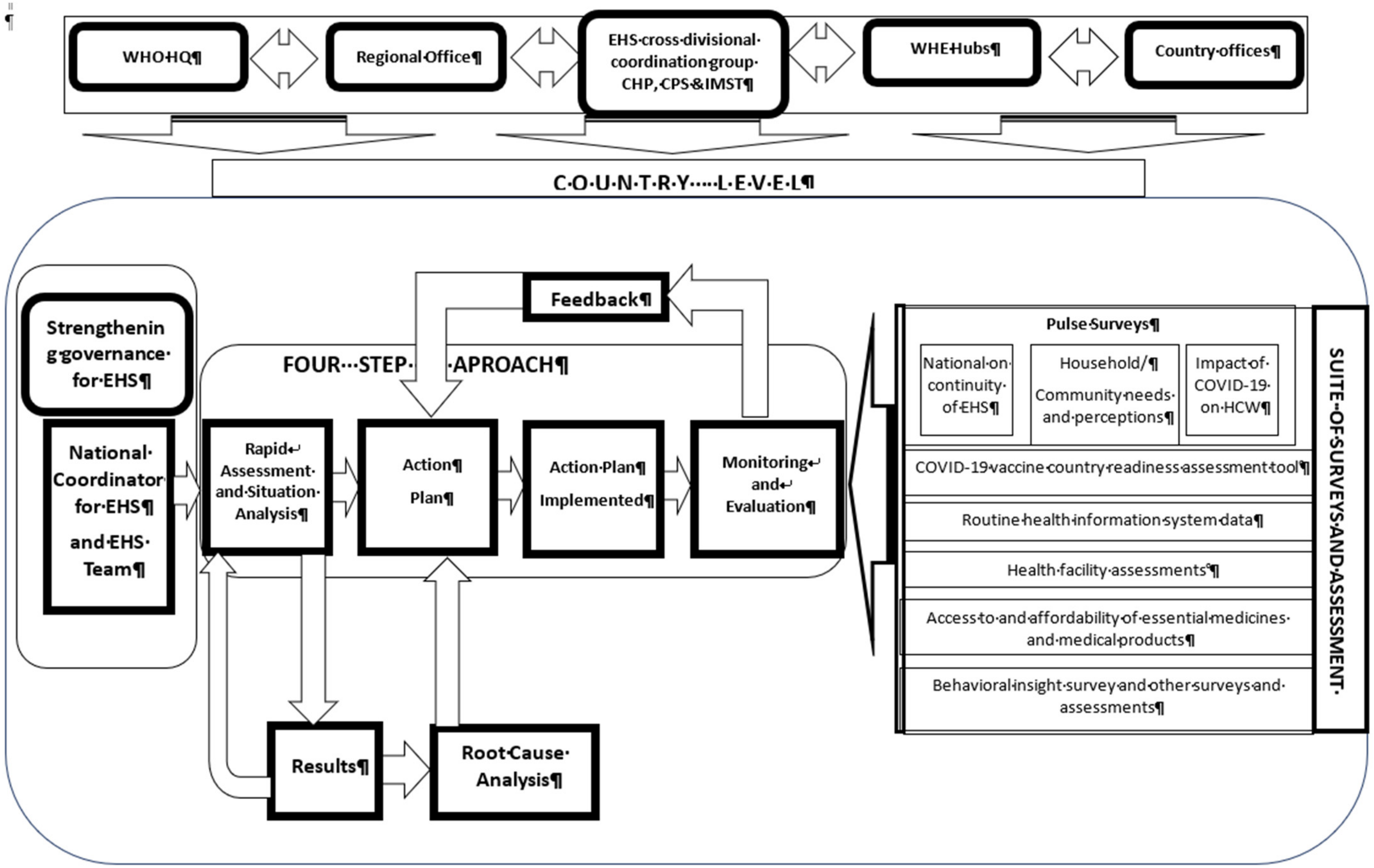

Supplement: Supplementary file 1 [file behavsci-12-00495-s001.zip › behavsci-1968302-supplementary.pdf]
